# TransCent: Computational enzyme design by transferring active sites and considering constraints relevant for catalysis

**DOI:** 10.1186/1471-2105-10-54

**Published:** 2009-02-10

**Authors:** André Fischer, Nils Enkler, Gerd Neudert, Marco Bocola, Reinhard Sterner, Rainer Merkl

**Affiliations:** 1 Institut für Biophysik und Physikalische Biochemie, Universität Regensburg, 93040 Regensburg, Germany; 2 Institut für Pharmazeutische Chemie, Universität Marburg, 35032 Marburg, Germany

## Abstract

**Background:**

Computational enzyme design is far from being applicable for the general case. Due to computational complexity and limited knowledge of the structure-function interplay, heuristic methods have to be used.

**Results:**

We have developed TransCent, a computational enzyme design method supporting the transfer of active sites from one enzyme to an alternative scaffold. In an optimization process, it balances requirements originating from four constraints. These are 1) protein stability, 2) ligand binding, 3) pK_a _values of active site residues, and 4) structural features of the active site. Each constraint is handled by an individual software module. Modules processing the first three constraints are based on state-of-the-art concepts, *i.e. *RosettaDesign, DrugScore, and PROPKA. To account for the fourth constraint, knowledge-based potentials are utilized. The contribution of modules to the performance of TransCent was evaluated by means of a recapitulation test. The redesign of oxidoreductase cytochrome P450 was analyzed in detail. As a first application, we present and discuss models for the transfer of active sites in enzymes sharing the frequently encountered triosephosphate isomerase fold.

**Conclusion:**

A recapitulation test on native enzymes showed that TransCent proposes active sites that resemble the native enzyme more than those generated by RosettaDesign alone. Additional tests demonstrated that each module contributes to the overall performance in a statistically significant manner.

## Background

Enzymes are highly specific and efficient biocatalysts. It is of great scientific and practical interest to alter the function and stability of enzymes, or even generate them *de novo *from first principles [1]. Due to our limited understanding of the structure-function interplay, most of the successful enzyme design examples have been achieved by "directed evolution", *i.e. *by performing several rounds of random mutagenesis in combination with efficient screening or selection systems to isolate beneficial variants [2]. However, the complexity of the problem calls for computational methods that are aimed at guiding enzyme design experiments, and during the last years we have seen significant progress along these lines. The first computer-based approaches described were Dezymer [3] and ORBIT [4]. Since then, novel program suites have been developed [5-7], and a few successful experimental enzyme designs have been based on computational methods [8,9]. Only recently, the successful design of Kemp elimination catalysts [10] and of retro-aldol enzymes accommodating a multistep reaction [11] have been reported. Despite this progress, computational enzyme design is far from being applicable to any arbitrary problem.

Why is this the case? The structural basis of enzyme catalysis is often not well understood, which makes it difficult to define the critical optimization criteria in a specific enzyme design problem [12]. Factors that might be relevant and often mutually interdepend are binding of the substrate and transition state of a given reaction, release of the product, conformational flexibility or dynamics, and pK_a _values of catalytic residues. Considering protein dynamics is still beyond the scope of current optimization methods. However, ligand-binding [13] and adjusting pK_a _values of active site residues [14] can be integrated into the optimization process. Additionally, a statistical analysis of homologous proteins might help to incorporate factors that cannot be considered explicitly. Multiple sequence alignments (MSAs) have already been used for consensus design approaches [15]. Ensemble-based scoring functions [16] and knowledge-based potentials [17] turned out to be valuable approaches for deducing additional characteristics from larger datasets.

Along these lines, we designed the computational enzyme design program TransCent. In order to focus on the most relevant determinants, we restrict the design goal to a well defined task: This is the transfer of an active center from one enzyme (the template) to a second protein (the scaffold) whose backbone remains fixed. The novel approach implemented with TransCent is the concurrent consideration of four constraints during modeling. These are protein stability, ligand binding, pK_a _values of active site residues, and structural features of the active site described by knowledge-based potentials. The program comprises four modules, each of which processes one constraint. We show that each module contributes to the quality of the 3D model, and that the combination of all modules performs best. As a first application, we present and discuss models transferring active sites of (βα)_8_-barrels, which form a frequently encountered and catalytically versatile enzyme family [18,19].

## Results

### Prerequisites and conventions

TransCent supports the transfer of an active site from one enzyme (the template) to another protein backbone (the scaffold). We focused on design problems that fulfill the following prerequisites: 1) The 3D structure of both the template and the scaffold are available with adequate quality (resolution < 2.5 Å, position of all atoms in the active site resolved, no loops missing). 2) The pose of the ligand-template complex is known. 3) The active sites of the scaffold and the template can easily be superimposed. 4) For the template, the sequences of at least 80 homologous proteins are available, allowing the inference of a well populated MSA.

Moreover, in order to reduce computational complexity of the algorithm, the following assumptions and restrictions are also effective: 5) The backbone of the scaffold and the position of the ligand are kept fixed in 3D space during optimization. 6) Only side chain conformations of a backbone dependent rotamer library [20] are considered. 7) The pose of the ligand as observed in the template is assumed to be the relevant one for catalysis (*i.e. *represents the active binding mode).

Note that the above limitations are either related to the precision required for reliable predictions (conditions 1 and 2) or could be circumvented by combining TransCent with existing programs like RosettaMatch [6] (condition 3) or by computing transition states [11] (condition 7). Computational complexity imposes conditions 5 and 6; for many proteins condition 4 can easily be accomplished due to the abundance of completely sequences genomes.

During the design process, residues are grouped with respect to their distance from the ligand. We name residues, which have to the ligand a distance of at most 7 Å, the active center *ACT_CENT*. *ACT_CENT *is surrounded by a shell *SHELL_1 *consisting of residues having a distance between 7 Å and 15 Å. All other residues belong to *SHELL_2*. The central shells are larger than those used elsewhere [11] in order not to miss relevant residues. In the design phase, the backbone of the scaffold has to be decorated with side-chains. Residues belonging to *ACT_CENT *will be redesigned; residues of *SHELL_1 *will be repacked to flexibly embed the amino acids constituting *ACT_CENT*. For *SHELL_2*, TransCent keeps residues and side chain conformations as found in the scaffold. Altogether, this selection of constraints is a compromise of speed and precision by allowing a flexible modeling of the active site and the conservation of remote residues, which are presumably less relevant for catalysis.

### A framework for enzyme design

A typical protein design program consists of three elements [21]. These are a modeling unit, which generates the atomic details of a protein model, a unit that evaluates the quality of a model *via *an energy function, and an optimization unit that directs the design process to find low energy configurations. A state-of-the-art program for protein modeling is RosettaDesign [22], which predicts an optimal sequence for a given backbone and comprises the above mentioned three elements. We utilized Rosetta as a framework for TransCent, *i.e. *we did not alter the modeling and optimization unit. However, we extended its energy function to include features relevant for enzyme design. The novel energy function consists of four terms, which are computed by separate modules. These are related to protein stability (ST-module), ligand binding (LB-module), knowledge-based potentials (KP-module), and pK_a_-values (PK-module). In the following, these modules are described in detail.

### Protein stability: ST-module

Rosetta's built-in energy function generates sequences to optimize protein stability by combining terms for van der Waals interactions, hydrogen bonds, solvation, a knowledge-based pair-wise potential that accounts primarily for electrostatics, and a score derived from the frequency of rotamers deposited in a library. The outcome of many studies has demonstrated the excellent performance of Rosetta for predicting stable proteins [23]. Therefore, to assess the stability of a protein model, we decided to use Rosetta's approach and named the corresponding energy term *E*_*ST*_:

(1) $$E_{ST}=\sum_{i=1}^{n}E(res_{i})+\sum_{i=1}^{n}\sum_{j=i+1}^{n}E(res_{i},res_{j})$$

*E*_*ST *_is the sum of all self-energies *E(res*_*i*_) and pairwise energies *E(res*_*i*_, *res*_*j*_) for all residues *i *and all residue pairs *i, j *of a 3D model [22]. In order to increase speed, Rosetta stores pre-calculated partial results of this energy term in tabular form. The ST-module accesses this table during the optimization process. As an alternative to Rosetta's approach, a different energy function like the one implemented by EGAD [7] might be utilized for the ST-module.

### Ligand binding: LB-module

A prerequisite for catalysis is substrate binding. Since many of the X-ray structures of the template enzymes do not have the true substrate bound but a substrate analogue/product/product analogue, we termed this module ligand-binding module. During modeling, three constraints have to be considered. These are 1) the positioning of the ligand, 2) ligand conformation and 3) adequate interactions of the ligand with the atoms of the scaffold making up the binding site. TransCent expects a specification of the ligand position and its conformation as input. It is the task of the LB-module to optimize the interaction of the active site with the ligand. For this purpose we utilized DrugScore, which is a knowledge-based scoring function for protein-ligand interactions [24]. Based on this potential, the LB-module computes a score *E*_*LB*_:

(2) $$E_{LB}=\sum_{i=1}^{n}S_{DrugScore}(res_{i})$$

*E*_*LB *_is the sum of DrugScore energies determined for the interaction of ligand atoms with residues of the model.

### Knowledge-based potentials: KP-module

For modeling enzyme function, it is crucial to parameterize all relevant aspects of the active site. However, frequently it is unclear, which details of a protein structure are relevant for catalysis. In such cases, the usage of knowledge-based potentials is a proper method of including information given implicitly by protein structures or sequences [25]. We utilized this approach to determine type and location of catalytic active residues and those residues that interact with the ligand by analyzing residue conservation and networks of hydrogen bonding (see Methods).

**Figure 1:**
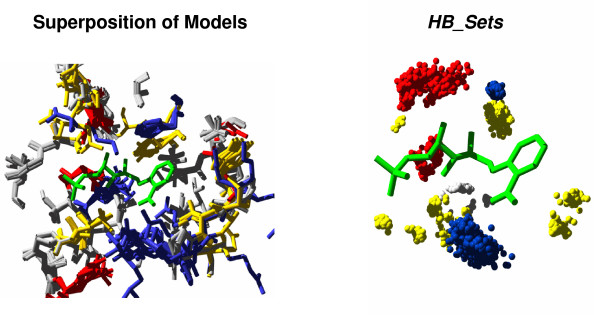
**Determination of knowledge-based potentials for a template's active site**. In order to illustrate the determination of potentials, 10 3D models were created for homologs of TrpF and superimposed (left panel). The right panel shows the 3D position of groups (the various sets *HB_Set*_*i*_) forming hydrogen bonds with the ligand rCdRP, which is plotted in green. The geometry of resulting scatter-plots was utilized to determine for each residue a knowledge-based potential; see Methods for details. Color code: (Arg, His, Lys), blue; (Asp, Glu), red; (Asn, Cys, Gln, Ser, Thr, Tyr), yellow. Hydrophobic residues are plotted in grey. Plots were generated by using SWISS-MODEL.

A prerequisite for the determination of knowledge-based potentials is a sufficiently large number of structures that can be exploited for statistical analysis. However, for enzyme design the number of highly resolved structures is too low in most cases. In order to increase the number of samples, we created homology models, which we utilized as a surrogate. Modeller [26] was fed with homologous sequences originating from the respective Pfam [27] entry to which the template belongs. We only used highly similar sequences during homology modeling: For residues constituting *ACT_CENT*, sequence identity with the template had to be > 40% and the average T-Coffee core index [28] (which indicates the quality of the alignment) had to be > 2.0. It is known that composition and 3D arrangement of active sites are generally highly conserved. Therefore, one can expect high precision for the predicted arrangement of residues participating in catalysis and may use the models alternatively to known structures. Using a superposition of these models and known structures, for each hydrogen bond involving ligand atoms, its variation in 3D position was determined and utilized to parameterize shape and location of an ellipsoid constituting an individual probability density function (*PDF*). These *PDF*s were transformed to knowledge-based potentials (*KBP*, see Methods) which formed the basis for the computation of the energy term *E*_*KP*_. Figure 1 illustrates our approach.

For each residue of an active site to be modeled, a set of atoms *PUT_HB_SET*_*i *_may participate in hydrogen bonds with the ligand; see Methods. For an assignment of individual *PUT_HB *atoms to *KBP*s, we used the Hungarian Method [29]. In each case, the outcome of an assignment are three sets: *HB_ KBP *consists of those *PUT_HB *atoms and *KBP*s assigned to each other. *HB_UNLINKED *contains all *PUT_HB *atoms that were not allocated to a *KBP*, and *KBP_UNLINKED *subsumes all *KBP*s, that were not occupied by a *PUT_HB *atom. Based on this assignment, *E*_*KP *_is computed as:

(3) *E*_*KP *_= *E*_*HB_KBP *_+ *E*_*HB_UNLINKED *_+ *E*_*KBP_UNLINKED*_

The three terms score those residues contributing to the related sets; see Eq. (13).

### Optimizing pK_a _values: PK-module

In many cases, pK_a _values of titratable groups belonging to the active site are shifted. These shifts can be crucial for catalysis if the groups participate in proton transfer steps. Therefore, the respective pK_a _values arising in a designed site have to be similar to those found in the template. The level of pK_a _perturbation depends on the local environment of the respective residue. This is why pK_a _shifts impose an additional constraint for sequence optimization. PROPKA [30] is one of the most accurate methods for computing pK_a _values [31]. However, although it is also a fast method, we had to further increase execution speed in order to incorporate pK_a _determination into the optimization process. The PK-module computes the energy term *E*_*PK*_:

(4) $$E_{PK}=\sum_{i=1}^{m}cons(res_{i})\cdot |pK_{a}(res_{i}^{template})-pK_{a}(res_{i}^{model}){|}^{\lambda_{pKa}}$$

Here, $pK_{a}(res_{i}^{template})$ is the predicted pK_a _value for residue *i *of the template used as a reference and $pK_{a}(res_{i}^{model})$ is the predicted pK_a _value of the corresponding residue in the model. The factor λ_*pKa *_was determined together with other parameters (see below). λ_*pKa *_= 2.0 turned out to be adequate, which is in agreement with previous recommendations [32]. The weight *cons(res*_*i*_) is related to residue conservation deduced from the amino acid frequency distribution of an MSA of homologous proteins; see Methods, Eq. (7). It is 1.0 for strictly conserved residues and decreases for less conserved ones. The assignment routine of the KP-module was utilized to deduce from the set *HB_ KBP *those *m *residues that were considered for pK_a _optimization.

### A combined energy function

Based on the outcome of the four modules, TransCent computes the energy *E*_*TransCent*_:

(5) *E*_*TransCent *_= 1.0·*E*_*ST *_+ *w*_*LB*_·*E*_*LB *_+*w*_*KP*_·*E*_*KP*·_+ *w*_*PK*_·*E*_*PK*_

which is a combination of the above introduced terms. We utilized the outcome of the ST-module as reference. Therefore, *w*_*ST *_is 1.0. The remaining three weight factors and λ_pKa _were determined by analyzing a training set (see below). TransCent performed best with *w*_*LB *_= 0.15·10^-3^, *w*_*PK *_= 0.5, and *w*_*KP *_= 1.0. *E*_*TransCent *_was embedded into Rosetta as an alternative energy function. Note that individual energy terms of *E*_*TransCent *_can be eliminated by setting the respective weight to 0.0. In the following, we will designate a non-standard combination of modules by enumerating active modules as for example in TransCent(ST, LB). In this case, the ST- and the LB-modules are enabled and the KP- and the PK-modules are disabled. The term TransCent(*) is equivalent to TransCent(ST, LB, KP, PK).

### Training the weights

A common method for *in silico *training and for benchmarking design algorithms is the recapitulation of native proteins [3,6,22,33], where – for a given set of examples – the concordance of calculated models and the wild-type is evaluated. We selected a set of 128 enzymes (see Materials), which we named *ENZ_TEST*. For training and evaluation, we used two different similarity measures, both of which assess the correspondence of protein sequences of a given model and the native protein. The first one [*IDENT_RES*] was the percentage of identical residues; the second one [*BLOSUM_SCORE*, see Eq. (6)] was deduced from amino acid similarity. Both values were determined by analyzing residues belonging to the active centers *ACT_CENT*. Among the proteins of *ENZ_TEST*, the size of active centers varied from 10 to 60 residues; the mean was 28 residues. Due to the simulated annealing protocol [34] used during optimization, final models may differ for individual experiments. Therefore, we computed for all entries of *ENZ_TEST *10 individual designs each and determined the mean for both similarity measures. In the following, these mean results will be reported.

At first, this approach was used to determine optimal weights required for Eq. (5). Different combinations of TransCent's modules were used and weights were varied within an appropriate range of values. Those weights were identified that gave the highest *BLOSUM_SCOREs*. The outcome of these computations was as follows:

#### ST-module

In order to assess the improvement gained by combining TransCent's modules, we first determined the performance of the ST-module on *ENZ_TEST*. The *IDENT_RES *values varied between 9% and 56%, the mean was 29.5%; the *BLOSUM_SCOREs *were between -0.4 and 3.4, the mean was 1.1. The rank correlation of *IDENT_RES *values and *BLOSUM_SCORE *values was high with statistical significance (*r*_*s *_= 0.92, *p *<< 0.001), indicating that both similarity measures are equally well suited to evaluate model quality.

#### LB-module

For the determination of *w*_*LB*_, the LB-module was combined with the ST-module. *IDENT_RES *and *BLOSUM_SCORE *values had an optimum at *w*_*LB *_= 0.15·10^-3^. In this case, the mean *IDENT_RES *value was 37% and the mean *BLOSUM_SCORE *was 1.5.

#### KP-module

A prerequisite for using this module is the existence of a sufficiently large set of homologous sequences, which was the case for the 27 elements of the subset named *ENZ_TEST*_*hom*_. To determine an optimal weight *w*_*KB*_, TransCent(ST, KP) was used. In this case, both the *IDENT_RES *values and the *BLOSUM_SCORE *values did not show a distinct optimum, but ascended a plateau. In order to avoid an overvaluation of the associated potential, we selected *w*_*KP *_= 1.0 which is the smallest value gaining the plateau. In this case, the mean *IDENT_RES *value was 48% and the *BLOSUM_SCORE *value was 2.2.

#### PK-module

This module depends on the identification of a specific set of residues accomplished by the KP-module (see Methods). Therefore, *ENZ_TEST*_*hom *_was analyzed using the combination TransCent(ST, KP, PK). As *w*_*KP *_was set to 0.0, the contribution of *E*_*KP *_was disabled. Performance was maximal for *w*_*PK *_= 0.5. In this case *IDENT_RES *was 39.5% and the *BLOSUM_SCORE *was 1.7.

### Assessing TransCent's performance

**Figure 2:**
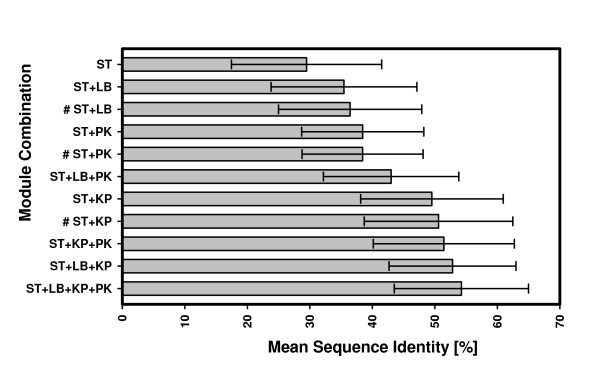
**The performance of different module combinations of TransCent as judged by *in silico *recapitulation of active sites**. Mean *IDENT_RES *values and standard deviations were determined for different combinations of modules. Abbreviations for modules are: ST (stability), LB (ligand binding), KP (knowledge-based potential), and PK (pK_a _values). For each combination of modules, 20 models were generated for each enzyme belonging to *ENZ_TEST*_*hom*_. Values labeled with a # originate from a leave-one-out cross validation test.

A central paradigm for evaluating the quality of a design program is the *in silico *recapitulation experiment introduced above. Due to the specific requirements of catalysis, active sites are generally highly conserved. Therefore, the comparison of modeled sites with the active site of the wild-type enzyme allows the evaluation of a program's performance. In order to assess the contribution of individual modules to the performance of TransCent, eight different combinations of TransCent's modules were tested. We generated 20 models for each enzyme belonging to *ENZ_TEST*_*hom*_. Mean *IDENT_RES *values were determined and plotted. Results are summarized in Figure 2. The data clearly show that each module contributes significantly to the performance of TransCent. Compared to an exclusive usage of the ST-module, the combination of all four modules resulted in an increase of identical residues from 29.5% to 54.3%. A *t*-test based on *IDENT_RES *values showed that each addition of a module improved the performance in a statistically significant manner (*p *<< 0.01).

One might argue that training and test data were not separated for the above evaluation. However, as we trained only four parameters (*w*_*LB*_, *w*_*KP*_, *w*_*PK*_, λ_*pKa*_), the program cannot memorize specific arrangements of active sites possessing hundreds of degrees of freedom each. In order to confirm this argument and to show that our training allows an unbiased determination of the above weights, we performed a leave-one-out test. Each of the 27 enzymes belonging to *ENZ_TEST*_*hom *_was used for validation, while deducing individual weights from the remaining 26 enzymes taken for training as described above. By conducting a grid search, optimal weight factors were determined. These factors were then used to measure the performance of each module in combination with the ST-module. Results were added to Figure 2. As can be seen, there is no significant performance difference between related tests. This result indicates that the training set allows a robust determination of the above parameters and confirms that the parameter set generalizes well.

**Figure 3:**
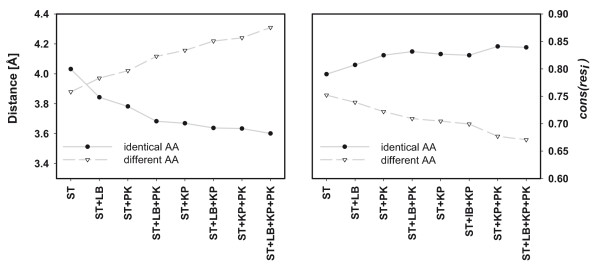
**Dependence of distance to the ligand and residue conservation for recapitulated and not recapitulated residues on TransCent's configuration**. Different combinations of modules were used to generate 20 models each in a recapitulation experiment for all enzymes belonging to *ENZ_TEST*_*hom*_. All residues *res*_*i *_ϵ*ACT_CENT *were analyzed. The left panel depicts the mean distance of their C_β_-atoms (C_α _in case of Gly) to the nearest atom of the ligand. The right panel depicts the mean conservation as deduced from the respective columns of the MSA and as expressed by the score *cons(res*_*i*_). For the models, residues were grouped: "identical AA" are those residues possessing the same amino acid as the templates, "different AA" are those ones, where TransCent proposed a different amino acid. Abbreviations for modules are: ST (stability), LB (ligand binding), KP (knowledge-based potential) and PK (pK_a _values).

For the above recapitulation test, all residues of *ACT_CENT *are considered equally important. However, their distance to the ligand and their conservation levels (deduced from the MSA) differ. For a model of higher quality, it is plausible to expect a higher rate of recapitulated amino acids at those positions which are closer to the ligand or which are more conserved. Analyzing these two parameters, we further assessed the performance of TransCent. Different combinations of modules were used to generate 20 models each in a recapitulation experiment for all enzymes belonging to *ENZ_TEST*_*hom*_. For each residue *res*_*i*_, the distance of the C_β_-atom (C_α _in case of Gly) to the nearest atom of the ligand was determined. In addition, related MSAs were used to deduce the residue-specific conservation *cons(res*_*i*_); see Eq. (7). Modeled residues were grouped according to their concordance with the template. The group "identical AA" contains the recapitulated positions; "different AA" are those ones, where TransCent proposed an amino acid not seen in the template. For these two groups, mean distance values were determined and plotted. Figure 3 shows the results. By utilizing more modules, the distance of "identical AA", *i.e*. recapitulated residues, decreased steadily from 4.0 Å when using TransCent(ST), to 3.6 Å when enabling all modules. Synchronously, the distance of "different AA" increased from 3.9 Å to 4.3 Å. That is, the more modules are used, the higher is the probability that residues located close to the ligand are recapitulated. The right panel depicts the mean conservation as deduced from the respective MSAs and as expressed by the score *cons(res*_*i*_). By using more modules, the conservation level of "identical AA" (recapitulated residues) increased, whereas the conservation level of "different AA" decreased. For TransCent(ST), the conservation for "identical AA" is 0.79 and for "different AA" it is 0.75. For TransCent(*), the mean conservation for "identical AA" increased to 0.84, whereas the score for "different AA" fell to 0.64. That is, the more modules are used, the higher is the probability of conserved residues to be recapitulated. In summary, the results indicate that the active sites became more similar to the template by using additional modules. Note that the shell defining an active site for TransCent is larger than that used elsewhere [11]. Therefore, it is not implausible, that a certain fraction of these residues differs from the template. This notion is supported by the above results: The mean conservation for the set "different AA", *i.e. *those residues decorated by TransCent with an amino acid not seen in the template is 0.64. This value indicates a substantial degree of variation even in MSAs which sample closely related homologs.

### Recapitulating the oxidoreductase cytochrome P450

**Figure 4:**
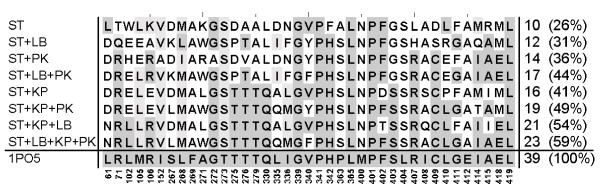
**MSA showing the outcome of *in silico *recapitulation experiments for cytochrome P450 2B4**. Different combinations of modules were used to generate models in a recapitulation experiment. The first column lists the module combination, the last column the number and fraction of correctly determined residues belonging to *ACT_CENT*. The last line gives the native sequence as deduced from pdb-entry 1po5. Recapitulated residues are indicated by a gray background. Abbreviations for modules are: ST (stability), LB (ligand binding), KP (knowledge-based potential) and PK (pK_a _values).

**Figure 5:**
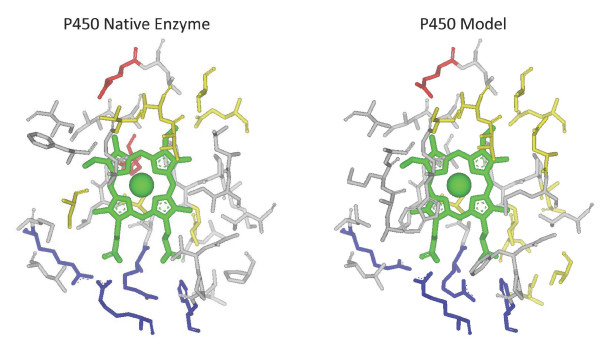
**Comparison of the residues constituting *ACT_CENT *in cytochrome P450 2B4 and a model generated in a recapitulation experiment**. In the left panel, the active site of cytochrome P450 (1po5) is plotted; in the right panel, the active site of the best model generated by TransCent is shown. The RMSD of the 23 recapitulated residues is 1.0 Å. Color code of side chains, which are depicted as sticks: (Arg, His, Lys), blue; (Asp, Glu), red; (Asn, Cys, Gln, Ser, Thr, Tyr), yellow. Hydrophobic resides are plotted in grey, the heme group is shown in green. Fig. 5 and 6 were created using Accelrys DS Visualizer 2.0 .

In order to exemplify the progress gained by combining TransCent's modules, we present the results of an *in silico *recapitulation which allows best to track the cooperation of the modules. Oxidoreductase cytochrome P450 2B4 (pdb code 1po5[35]) is – according to the SCOP classification [36] – an all-alpha protein. In this case, *ACT_CENT *consists of 39 residues. TransCent(*) recapitulated 23 (59%), whereas the ST-module alone recapitulated only 10 (26%) of the native residues. Figure 4 shows an MSA of *ACT_CENT *residues listing the proposals of the various combinations. There are only two positions (61 and 340), which were recapitulated by different module combinations but not by TransCent(*). At position 340, the module combinations TransCent(ST), TransCent(ST, LB), and TransCent(ST, LB, KP) proposed the native valine, all other combinations predicted a tyrosine or a phenylalanine. At position 61, only TransCent(ST) proposed the native lysine, all other combinations proposed an aspartic acid or an asparagine. In contrast, TransCent(ST) as well as TransCent(ST, LB) did not recapitulate a group of three arginine residues (positions 71, 106, 407). TransCent(ST, KP) and TransCent(ST, KP, PK) proposed two arginines and a glutamate, which constitute a salt bridge. Only after adding the LB-module, all three arginines were proposed. A further indicator for the high quality of the design is the similarity between the model and the native active site: A superposition of the 23 recapitulated side chains with the template gave an RMSD-value of only 1.0 Å for all side chain atoms (Figure 5).

### A case study: converting (βα)_8_-barrels that bind ribulose-phosphate

As a first application, *in silico *transfer experiments were performed with five enzymes belonging to the SCOP [36] superfamily of ribulose-phosphate binding proteins, which belong to the frequently encountered and catalytically versatile enzyme class of (βα)_8_-barrels [18,19]. These were the enzymes phosphoribosyl-5-amino-1-phosphoribosyl-4-imidazolecarboxamide isomerase (HisA), the cyclase subunit of imidazole glycerol phosphate synthase (HisF), phosphoribosylanthranilate isomerase (TrpF), indole-3-glycerol-phosphate synthase (TrpC), and the α subunit of tryptophan synthase (TrpA). HisA and HisF catalyze two successive steps within histidine biosynthesis, whereas TrpF, TrpC, and TrpA catalyze three consecutive reactions within tryptophan biosynthesis [37]. For HisA and HisF, the ligand is *N*1-[(5'-phosphoribosyl)formimino]-5-aminoimidazol-4-carboxamide ribonucleotide (PRFAR). Reduced 1-(*o*-carboxyphenylamino)-1-deoxyribulose 5-phosphate (rCdRP) is bound to TrpF and TrpC; imidazole glycerol phosphate (IGP) is bound to TrpA. Note that the ligand of HisA and HisF, which contains two sugar phosphate moieties, is twice as large as the ligands of TrpF, TrpC and TrpA, which contain only a single sugar phosphate group. Although the general topology of these five enzymes is similar, the mean RMSD value of related C_α_-atoms is as high as 3.0 Å, as determined by TM-align [38]. For each of the experiments described below, 10 models were generated and mean similarity values were calculated. Based on the five enzymes, 25 models can be generated; among these are five recapitulation experiments. For the latter cases, the mean number of identical residues in active sites was between 46% and 54%, which is in agreement with the mean performance of the program (see Figure 2). For the transfer experiments, the mean sequence identity value was 27%. In the following, several models resulting from recapitulation and transfer experiments are described.

**Figure 6:**
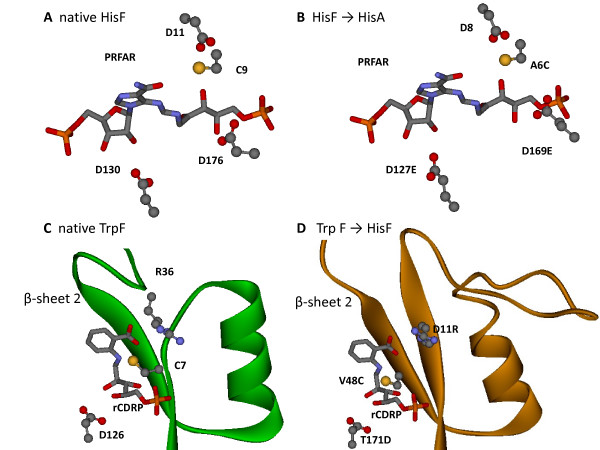
**Comparison of wild-type active sites with transfer models HisF(template) → HisA(scaffold) and TrpF(template) → HisF(scaffold) determined by TransCent**. The catalytic residues in native HisF (A, 1thf) and the designed residues of the HisF → HisA transfer (B) are shown in complex with PRFAR (HisF substrate and HisA product). The designed residues in the HisA scaffold are located at equivalent positions as in the HisF template (compare panel B and A). The catalytic residues in native TrpF (C, 1lbm) and the corresponding residues of the designed TrpF → HisF model (D) are shown in complex with rCdRP (product analogue of TrpF). In wild-type TrpF, Cys 7 is located on β-sheet 1, and Arg 36 is located on a long loop after β-sheet 2. In the model, an arginine residue, which is responsible for ligand binding, is placed in the elongated β-sheet 1 (D11R exchange), whereas the catalytic cysteine (V48C exchange) is located in β-sheet 2. The aspartic acid D126 (TrpF) and the designed exchange T171D (TrpF → HisF transfer) are located at equivalent positions.

HisA (1qo2[39]) and HisF (1thf[39]) are similar, both with respect to structure and function. The product of HisA is the substrate of HisF; moreover HisF of *Thermotoga maritima *has weak HisA activity [39]. Three aspartate residues are strictly conserved among and between the HisA and HisF enzymes (Asp 8, Asp 127, Asp 169 in HisA; Asp 11, Asp 130, and Asp 176 in HisF). These aspartates are either essential or important for turnover of the substrates (ProFAR in case of HisA, PRFAR in case of HisF); additionally, a conserved threonine residue (Thr 161 in HisF; Thr 164 in HisA) influences enzymatic activity [40,41]. TransCent's HisF recapitulation restored at their correct position all above mentioned residues as well as Cys 9, which is also conserved in the known HisF sequences (Figure 6A). This finding indicates that TransCent was able to reconstruct the catalytic environment of the PRFAR ligand guided by the knowledge-based HB potential energy *E*_*KP*_. (The binding mode of PRFAR in HisF was taken from the X-ray structure of the yeast enzyme, 1ox5[42]. In agreement with these findings, TransCent also chose these residues or chemically similar ones (D127E and D169E exchanges) for the HisF(template) → HisA(scaffold) transfer (Figure 6B). The comparison of the *E*_*ST *_scores computed for the HisF recapitulation and the HisF → HisA transfer signaled only a minor loss of protein stability.

Recapitulating HisA is hindered by insufficient data, as no X-ray structure of HisA in complex with a bound ligand is known. Alternatively, we used for the design process HisA with modeled PRFAR. The complex of HisA and PRFAR was minimized employing the force field MAB as implemented in the Program Moloc [43]. An inspection of the HisA recapitulation and the HisA → HisF design showed that TransCent introduced the mutations D8K during recapitulation and D11K in the transfer experiment, thus replacing one of the negatively charged catalytic aspartates by a positively charged lysine residue. The Rosetta energy score *E*_*ST *_indicates a stabilization of about -1 kcal/mol caused by this exchange. In addition, the catalytically important Thr 164 was replaced by leucine during recapitulation. In the modeled binding mode the distances of residues Asp 8 and Thr 164 to the ligand are above TransCent's global cutoff (4.0 Å); consequently no knowledge-based potential was determined. Therefore, amino acids were selected upon stability criteria for these positions. This is why the HisA → HisF transfer sequence contains the exchanges D11K and T171A, although the native HisF residues would match the catalytic residues Asp 8 and Thr 164 in HisA. The positively charged lysine side chain of D11K is placed near the negatively charged D176E on the opposite side of the active site, enabling a stabilizing electrostatic interaction. This example indicates that the visual inspection of the models is crucial to detect stabilizing mutations, which may be disadvantageous for catalysis and could be due to parameter settings or limitations of our approach.

TrpF catalyses the isomerization of phosphoribosylanthranilate (PRA) to carboxyphenylamino-1'-deoxyribulose-5'-phosphate (CdRP). The X-ray structure of TrpF (1lbm[41]) from *T. maritima *in complex with the product analog rCdRP is known; the catalytic residues are Cys 7 and Asp 126 [41]. TransCent's redesign of TrpF recovered the complete environment around the ligand, including the catalytic residues (Figure 6C). For the 41 residues making up *ACT_CENT*, the program proposed 17 exchanges; 11 are located in loops at the surface. The remaining 6 replacements Q81N, A103G, L124T, V155I, S135T, and V179A, which are located inside the barrel, are conservative ones. Energy minimization of the crystal structure and the model resulted in similar stability and ligand binding energy.

When judging the quality of a model with respect to the successful recapitulation of catalytic residues and calculated ligand binding energy, then the best design of the TrpF template was accomplished on the HisF scaffold. It has been shown experimentally that moderate TrpF activity can be established on the HisF-scaffold by mutating Asp 130 to a non-negatively charged residue [44]. In our TrpF → HisF transfer, TransCent proposed the D130H exchange, reconstituting His 83 of the TrpF template. The essential catalytic residues Cys 7 and Asp 126 of the template were introduced in the scaffold by TransCent at the correct locations near the ligand by exchanges V48C and T171D (Figure 6C, D). Interestingly, Asp 126 and T171D are both located on β-sheet 6 and perfectly superimpose, whereas Cys 7 is located on β-sheet 1, but V48C on β-sheet 2. This finding demonstrates that TransCent can position an essential residue in a different secondary structure element of the scaffold than utilized in the template. This capability is further demonstrated by an arginine interacting with the anthranilic acid moiety of the ligand: In the TrpF template, Arg 36 is located in the long loop after β-sheet 2 and its positively charged guanidinium side chain forms a salt bridge with the negatively charged carboxylate moiety of rCdRP (Figure 6C). In the HisF scaffold this loop is missing. However, β-sheet 1 is elongated and the exchange D11R introduces an arginine with a side chain oriented towards the ligand (Figure 6D). In this case, all three terms *E*_*LB*_, *E*_*KP*_, and *E*_*PK *_are negative, *i.e. *all constrains demand for this mutation. In summary, the above examples illustrate the interplay of TransCent's modules when constructing active sites.

## Discussion

### Computational enzyme design methods improve slowly and are far from being perfect

Recent work [10,11] demonstrates that the *de novo *design of enzymatic activities not found in natural biocatalysts has become feasible. However, the designed activities were considerably lower than those evolved by nature [45], although the computation consumed more than 100 000 CPU hours. Thus, in spite of these pioneering efforts, we are still far away from an adequate understanding of enzyme structure-function relationship and the *de novo *design of highly effective active sites.

When developing TransCent, we followed a less ambiguous goal by transferring an already existing active site to a different backbone. TransCent is based on RosettaDesign and comprises modules for the optimization of ligand binding (LB-module) and pK_a _values of essential residues (PK-module). Using knowledge-based potentials as implemented in the KP-module contributed favorably to the performance of TransCent, demonstrating that active site "fingerprints" can be deduced from homology models and structure databases. In order to limit computer time, we concentrated on the most important features of enzyme catalysis, accepting restrictions such as fixed backbones and ligands. However, due to the modular concept of TransCent and the structure of its energy function [Eq. (5)], additional constraints can easily be integrated. Along these lines, methods for assessing side-chain conformational entropy have been proposed recently [46] and the latest version of Rosetta contains a "Backrub"-model introducing local backbone flexibility [47].

### Approaching specific requirements in enzyme design

The modules of TransCent constitute an approach of a multi-objective optimization for enzyme design. Here, we utilized a classical energy function [Eq. (5)]; alternative non-standard approaches have recently been described [48]. Each of TransCent's modules contributes to the quality of the design and has its specific strengths and weaknesses that will be discussed below. In general, the ultimate proof demonstrating success in model building is the biochemical characterization of enzyme function. Unfortunately, wet-lab experiments are time-consuming and expensive. Therefore, *in silico *methods must serve as a surrogate especially for the evaluation of algorithms. However, these approaches allow us at best to demonstrate the plausibility of a design, and the assessment rests on the assumption that wild-type sequences are optimal for catalysis [22]. Trusting in this postulate, we have performed active center recapitulation experiments to estimate the significance of individual modules for the design success. It is the aim of the ST-module to guarantee the stability of the modeled protein. When used exclusively, this module recovered on average 30% of the wild-type residues (Figure 2). This value is within expectation for two reasons: 1) Active sites resemble more the surface than the core of a protein. For remodeling, a recovery rate varying between 27% for surface positions and 52% for core positions has been reported [22]. 2) Residues of the active sites often do not contribute to stability, and catalytically relevant residues even tend to destabilize the enzyme [49]. Therefore, programs focusing on protein stability will fail to recover these residues. The same holds for residues that are constrained due to the shape of the binding pocket. The observation that RosettaDesign nevertheless recovered almost one third of the residues in active sites could indicate that the backbone conformation restricts the selection of amino acids at certain positions. Differences in rotamer frequencies for backbone independent and backbone dependent libraries support this notion. In addition, recent studies indicate that side-chain rotamers may lock the backbone into slightly different conformations [47].

The LB-module aims at optimizing ligand binding. It uses a rotamer-based version of DrugScore allowing TransCent to assess the impact of each individual rotamer. However, due to algorithmic complexity, the rotamer-based version, in contrast to the original version, is not able to consider the desolvation effect, as the orchestration of neighboring residues influences the outcome markedly. Despite this restriction, the performance data shown in Figure 2 illustrates that the LB-module contributes significantly to the recapitulation success of TransCent.

The KP-module determines the characteristics of the template's active site by deducing potentials without expert knowledge. The module aims at optimizing the protein-ligand hydrogen bond network by arranging donors and acceptors in a way resembling the template. As the module considers residue conservation as well, the selection of amino acids can be carefully balanced. In other programs, relevant amino acids have to be fixed before active site optimization can be started and therefore only residues relevant for catalysis could be considered until now [3,6]. Thus, our approach of using knowledge-based potentials adds flexibility not yet implemented in traditional computational design methods. This option requires that the sequences of at least 80 homologous proteins must be available to deduce the potentials with acceptable quality. However, the sequencing of hundreds of genomes [50] during the past years ensures that this condition can be fulfilled in most cases.

The PK-module optimizes the electrostatic embedding of residues relevant for catalysis. We introduced this module, because in general it is not sufficient to merely place active site residues in the correct orientation. In addition, often a proper pK_a _value will be essential for a specific residue to act as general acid or base during catalysis. In accordance with this statement, the markedly improved activity of a computationally designed enzyme by means of a directed evolution experiment has been explained with the pK_a _shift of a catalytic site [10]. We consider the simultaneous optimization of protein stability and of pK_a _values in a rotamer based protein design framework as a key feature of TransCent. However, one might argue that ignoring the effect of ligand atoms onto pK_a _values or an insufficient accuracy of pK_a _value determination by PROPKA could render the results ambiguous. However, a systematic deviation of absolute values has only a minor effect on the outcome of the design, as both the template and the model are treated in the same way. In accordance with this notion, Figure 2 illustrates the significant positive contribution of the PK-module to the performance of TransCent.

### Transfer experiments will promote computational enzyme design

Reflecting the *status quo *of computational protein and enzyme design, the current potential and limitations of these methods become obvious. For several algorithms, their ability of creating stable proteins by decorating native backbones has been successfully demonstrated [51,52]. Therefore, generating *in silico *a stable protein based on a native fold should be feasible in most cases. For enzyme design, taking this constraint is clearly not sufficient and additional features such as substrate binding [13] and transition state stabilization [10] have to be considered to generate native-like active sites. Drafting artificial folds might be regarded as a further step towards *de novo *enzyme design. However, it is doubtful whether this effort is a necessary prerequisite to establish novel functions or to surpass the proficiency of existing enzymes. For example, when searching a suitable scaffold for the Kemp-elimination reaction, more that 100 000 locations for putative active sites were identified in natural folds [10]. In addition, the observed preference for the ancient and frequently encountered (βα)_8_-barrel [53], which accommodates enzymatic reactions covering five of the six classes defined by the Enzyme Commission [19], suggests that computational design can readily use folds evolved by nature. Nevertheless, in spite of first promising success cases, our limited understanding of most enzymatic reactions makes *de novo *design a very difficult task and leaves room for simpler, nevertheless instructive approaches. Following recapitulation experiments, the transfer of an existing active site to a new scaffold – as supported by TransCent – is the next obvious step to take. Both the *in silico *analysis of generated 3D models as done above, and particularly the biochemical characterization of the designed proteins will identify properties that were modeled in an acceptable or insufficient way. These findings will help to validate or improve in a feedback-loop [45] both TransCent as a whole and the individual methods implemented in its modules. Thus, transfer experiments will contribute to our understanding of enzyme function and bring forward computational enzyme design.

## Conclusion

TransCent is a computational enzyme design program, which predicts mutations in a scaffold aimed at establishing the activity of a template enzyme. During the design process, protein stability, substrate binding, pK_a _values of essential residues and knowledge-based hydrogen bonding networks are considered simultaneously by integrating separate optimization modules. Our *in silico *evaluation demonstrated that TransCent can recapitulate a considerable fraction of active site residues for a given template. We will now experimentally test some of the transfer designs in order to further judge the prediction quality of the program. Depending on the outcome, we will take advantage of TransCent's modular character to incorporate additional features such as backbone and ligand flexibility, which promises a further fine-tuning of the designed active site.

## Methods

### Test data *ENZ_TEST*

Using the following rules, 128 entries of the pdb database [54] were selected with a culling server [55]: 1) The resolution had to be at least 1.6 Å and the R-factor at most 0.25. 2) The structure had to be determined *via *X-ray crystallography and the sequence had to consist of at least 100 residues. 3) One ligand consisting of more than 10 atoms had to be part of the structure. 4) At least ten residues had to be not more than 5 Å apart from the ligand. 5) For the pairwise comparison of all entries, a maximal sequence identity value of 20% was tolerated. We named this set of 3D structures *ENZ_TEST*; additional file 1 lists the pdb codes. For maximal performance of TransCent, a set of at least 80 homologous sequences has to be available for a protein. This was the case for those 27 proteins printed bold in the data set listed in additional file 1. We named this set *ENZ_TEST*_*hom*_.

### Multiple sequence alignments

To create an MSA, we realigned the sequence of the template with sequences originating from the corresponding Pfam entry by using MAFFT [56]. Those sequences were selected that fulfilled two criteria imposed on residues belonging to *ACT_CENT *(For a definition of the set *ACT_CENT*, see Results). 1) A pairwise comparison with corresponding residues of the template resulted in a sequence identity value > 40%. 2) The mean T-Coffee core index [28] for these residues was > 2. This cut-off assures a sufficient quality of the alignment. We deduced these criteria from a recapitulation experiment: By using Modeller version 8.2 [26], we created homology models for at least 80 sequences and determined the mean RMSD value for all atoms of *ACT_CENT *residues of the template and the models. When applying the above criteria, the average RMSD was ~2 Å (determined by TM-align [38], data not shown).

### BLOSUM score

For the comparison of two sequences *A = a*_1_... *a*_*n*_, and *B *= *b*_1_... *b*_*n*_, we determined a mean BLOSUM score by computing

(6) $$BLOSUM\_SCORE(A,B)=\frac{1}{n}\sum_{i=1}^{n}BLOSUM_{62}(a_{i},b_{i})$$

As the sequences *A*, *B *of native proteins or models are of equal length, an alignment consists of *n *residue pairs making up sequences *A *or *B*. BLOSUM_62 _values are from the related scoring matrix [57].

### *Cons*(*res*_*i*_): Scoring the conservation of individual residues

To score the conservation of amino acids at an individual position *i *in an MSA, the following term [58] was computed:

(7) $$cons(res_{i})=\frac{2}{n/(n-1)}\sum_{j=1}^{n}\sum_{k=1}^{n}\frac{BLOSUM_{62}(as_{j}^{i},as_{k}^{i})}{BLOSUM_{62}(as_{j}^{i},as_{j}^{i})BLOSUM_{62}(as_{k}^{i},as_{k}^{i})}$$

*n *is the number of lines in the MSA, $as_{j}^{i}$ and $as_{k}^{i}$ are the amino acids occurring in lines *j *or *k *at position *i*. BLOSUM_62 _values are from the related scoring matrix [57]. For strictly conserved residues, *cons(res*_*i*_) is 1.0.

### Creating a superposition of models and structures

In order to increase the number of structures utilized for the determination of knowledge-based potentials, homology models were created. Starting from the respective Pfam [27] entry to which the template belongs, an MSA was generated (see above). Related sequences were fed into Modeller version 8.2 [26]. Resulting structures were superimposed based on the alignment given by the MSA. We name a template-specific ensemble of superimposed 3D structures *TEMPL_ENS*. For illustrations, plots were generated by using SWISS-MODEL [59].

### Setting the position and conformation of the ligand

For the transfer experiments described in Results, the input for the LB-module was generated by first superimposing active site residues of the template and the scaffold, and by transferring the ligand's pose from the template to the scaffold. In general, strategies developed for ligand docking [60] or drug discovery [61] as well as methods identifying key residues [62] may be considered for specifying the pose of the ligand.

### Determining knowledge-based potentials and scores

In order to establish a hydrogen bonding pattern for an active site, which is in agreement with those ones observed in *TEMPL_ENS*, knowledge-based potentials [25] were used. To specify these potentials, each residue position *i *belonging to *ACT_CENT *was considered separately. For each residue *i*, all atoms *HB *participating in hydrogen bonds with the ligand were determined. Candidates are those nitrogen, oxygen or sulphur atoms belonging to the side chains of Arg, Asn, Asp, Cys, Glu, Gln, His, Lys, Ser, Thr, Tyr, and Trp, which are in close proximity to the ligand (distance < 4.0 Å). Backbone atoms were not considered as the design algorithm does not alter the backbone conformation. All *HB *atoms related to position *i *were combined in the set *HB_SET*_*i*_.

For each set *HB_SET*_*i*_, a knowledge-based potential *KBP*_*i *_was deduced as a log-odds ratio of probability density functions [25]. The 3D positions of all atoms belonging to *HB_SET*_*i *_were used to determine a probability density function *PDF*_*obs*_, _*i *_modeled by means of a multivariate Gaussian distribution. By limiting in each direction the spread of a *PDF *to ± 3 σ from its center, it describes a volume with an ellipsoid shape. We named this volume *PDF_VOL*_*i*_. The expected probability function *PDF*_*exp*, *i *_is approximated as a uniform distribution filling *PDF_VOL*_*i*_. For each 3D coordinate *coord*, the corresponding probability was deduced from the *PDFs *in a cube of 1 Å ^3^. The knowledge-based potential $KBP_{i}^{3D}$(*coord*) is then defined as:

(8) $$KBP_{i}^{3D}(coord)=-\text{ln}\left(\frac{PDF_{obs,i}(coord)}{PDF_{exp,i}(coord)}\right)$$

Additionally, we added a factor that scores the conservation of those amino acids contributing to *HB_SET*_*i*_. The observed frequency *f*_*obs*, *i*_*(as) *is the number of cases where the amino acid *as *contributed to *HB_SET*_*i *_divided by #*TEMPL_ENS*, which is the number of structures. In order to determine the expected frequency *f*_*exp*, *i*_*(as)*, all rotamers of a backbone dependent library [20] were modeled at position *i*. *f*_*exp*, *i*_*(as) *results from the library-specific frequencies of *as *rotamers possessing a *HB *at position *i*. The potential was computed as

(9) $$KBP_{i}^{cons}(as)=-\text{ln}\left(\frac{f_{obs,i}(as)}{f_{exp,i}(as)}\right)$$

Scores (8) and (9) were combined to score positions originating from a model:

(10) $$S_{HB\_KBP_{i}}(res_{j})=KBP_{i}^{3D}(coord_{j})+KBP_{i}^{cons}(as_{j})$$

For modeling, we use *PUT_HB*_*j *_to name an atom of *res*_*j *_that might contribute a hydrogen bond with the ligand. An atom *PUT_HB*_*j *_has the coordinates *coord*_*j *_and belongs to an amino acid *as*_*j*_. Thus, it can be tested to what extent this *PUT_HB*_*j *_matches the preferences seen in *TEMPL_ENS*.

Not all hydrogen bonds occur in all structures of *TEMPL_ENS*. This indicates that some areas of the active site are indifferent with respect to polarity. To score these variations, a "hydrophobic tendency" was computed for each *PDF_VOL*_*i *_as a log-odds ratio:

(11) $$S_{apolar}(i)=-\text{ln}\left(\frac{f_{apolar,obs}(i)}{f_{apolar,exp}(i)}\right)$$

*f*_*apolar*, *obs*_*(i) *is the ratio of cases where no hydrogen bond was observed in structures of *TEMPL_ENS *divided by #*TEMPL_ENS*. Similarly, *f*_*apolar*, *exp*_*(i) *was deduced from the number of rotamers that cannot provide a *HB *atom by normalizing with the total frequency of rotamers for position *i *in the rotamer library. *S*_*apolar*_*(i) *is used to score cases where an active site of a model does not provide a *PUT_HB *atom that corresponds to the potential $KBP_{i}^{3D}$.

A fixed penalty *Penalty_Val *= 6.9 (deduced from an error rate of approximately 1/1000) was used to score *PUT_HB*_*j *_atoms of the model that could not be assigned to any *KBP*.

(12) *S*_*NO_HB *_= *Penalty*_*Val*

During optimization, the KP-module computes the energy *E*_*KP *_as a combination of three terms and based on the outcome of the assignment due to the Hungarian Method [29]:

(13) *E*_*KP *_= *E*_*HB_KBP *_+ *E*_*HB_UNLINKED *_+ *E*_*KBP_UNLINKED*_

*E*_HB_KBP _is the sum of *S*_HB_KBP _scores [see Eq. (10)] deduced from the model under study:

(14) $$E_{HB\_KBP}=\sum_{j}S_{HB\_KBP_{HM(res_{j})}}(res_{j})$$

The function *HM(res*_*j*_) selects the specific *KBP*_*i *_assigned to *res*_*j *_by means of the Hungarian Method. Here, all *PUT_HB *atoms are considered that belong to the current set *HB_KBP*. See also the description of the KP-module in Results.

The term *E*_HB_UNLINKED _originates from the number of unlinked *PUT_HB *atoms multiplied with the penalty score *S*_NO_HB_:

(15) *E*_*HB_UNLINKED *_= *#PUT_HP*_*UNLINKED*_·*S*_*NO_HB*_

Each *KBP *belonging to *KBP_UNLINKED *indicates the absence of a hydrogen bond in a certain region of the model. The energy *E*_KBP_UNLINKED _sums up *S*_*apolar*_*(res*_*i*_) values [see Eq. (11)] of these cases:

(16) $$E_{KBP\_UNLINKED}=\sum_{i}S_{apolar}(i)$$

## Authors' contributions

AF designed, implemented, and tested the software. NE participated in software development. GN prepared a modified version of DrugScore. MB accounted for expertise in molecular modelling and evaluated the models. RS contributed in biochemical expertise and assisted in manuscript writing. RM conceived of the approach, coordinated the project, and drafted the manuscript. All authors read and approved the final manuscript.

## Supplementary Material

Additional file 1 **Composition of data sets *ENZ_TEST *and *ENZ_TEST*_*hom*_.** The table lists the pdb-code of the proteins constituting the sets *ENZ_TEST *and *ENZ_TEST*_*hom*_. Click here for file
