# Supplementary material for: TransCent: Computational enzyme design by transferring active sites and considering constraints relevant for catalysis
Source: BMC Bioinformatics. 2009 Feb 10;10:54. doi: 10.1186/1471-2105-10-54 (PMC2667513; doi:10.1186/1471-2105-10-54)
Supplement: Additional file 1 — Composition of data sets ENZ_TEST and ENZ_TESThom. The table lists the pdb-code of the proteins constituting the sets ENZ_TEST and ENZ_TESThom. [file 1471-2105-10-54-S1.doc]

## Composition of *ENZ_TEST* and *ENZ_TEST_hom_.*

**128 structures were selected from the pdb-database according to the rules described in Methods. For these entries, constituting the set *ENZ_TEST*, their pdb-code is given. For optimal performance of TransCent, the sequences of at least 80 homologous proteins have to be on-hand for each structure. This is the case for those 27 entries printed in bold. This set was named *ENZ_TEST_hom_*.**

1a4i 1a6m **1ajs** **1b8o** 1ccw 1d2s 1d4o **1dbt** **1dqx** 1ds1 1dzk 1eix **1f74** **1f8e** 1f9v **1f9y** 1fp2 1fs7 1ft5 1g3p **1g6s** 1ghe 1gwe **1h4g** 1hqs 1hx0 1jcm 1jfb 1jub 1jx6 1k3y 1kly **1km4** **1kqp** 1kt6 1lbf **1lbm** 1ltz 1lyc 1m0k 1m15 **1m40** 1me4 1n08 1n8k 1nox **1o08** **1o8b** 1obd **1obo** 1ox5 **1po5** 1q0r 1q6z 1q92 1qnr **1qop** 1qwo 1qxy 1r2q 1r5l 1rcq 1rp0 1rwh 1rya 1s1d 1sg4 1si6 1su8 1t2d 1tbf 1tjy 1tt8 1u0f 1u4b **1u7g** 1uas **1ucd** **1ujp** 1usc **1v2x** 1vk5 **1vyr** 1w0h 1w0p 1w66 1w6g 1wb4 1wbe 1wdd 1wqw **1wui** 1wvf 1wvq 1x7d 1x8q 1x9i 1xdn 1xg0 1xg4 **1y0y** 1y3n 1ymt 1z2n 1z53 1z6f 1zdy 1zhx 1zk4 1zr6 1zz1 2a50 2a84 **2aeb** 2apj 2asb 2b82 2bfd **2bkx** 2bln 2bog 2bzg 2c1v 2czl 2f5t 2f6u 2nlr 2tys
